# Cell localisation of gadolinium-based nanoparticles and related radiosensitising efficacy in glioblastoma cells

**DOI:** 10.1186/s12645-014-0006-6

**Published:** 2014-10-10

**Authors:** Lenka Štefančíková, Erika Porcel, Pierre Eustache, Sha Li, Daniela Salado, Sergio Marco, Jean-Luc Guerquin-Kern, Matthieu Réfrégiers, Olivier Tillement, François Lux, Sandrine Lacombe

**Affiliations:** Institut des Sciences Moléculaires d’Orsay (UMR 8214) Bât 351, Université Paris Sud, CNRS, 91405 Orsay Cedex, France; Institut Curie, centre de recherche, bat 112, Centre Universitaire, 91405 Orsay Cedex, France; INSERM U759, bat 112, Centre Universitaire, 91405 Orsay Cedex, France; Synchrotron SOLEIL, BP48 Saint-Aubin, 91192 Gif-sur-Yvette, France; Institut Lumière Matière, Université Claude Bernard Lyon 1, CNRS, 69622 Villeurbanne cedex, France

**Keywords:** Radiosensitisation, Nanomedicine, Tumor targeting, Theranostic, Gadolinium- based nanoparticles, Localisation, Deep-UV synchrotron microscopy, Glioblastoma

## Abstract

Recently, the addition of nanoparticles (NPs) has been proposed as a new strategy to enhance the effect of radiotherapy particularly in the treatment of aggressive tumors such as glioblastoma. The physical processes involved in radiosensitisation by nanoparticles have been well studied although further understanding of its biological impact is still lacking, and this includes the localisation of these NPs in the target cells. Most studies were performed with NPs tagged with fluorescent markers. However, the presence of these markers can influence the NPs uptake and localisation. In this study, a set of methods was used to unambiguously and fully characterise the uptake of *label-free* NPs, their co-localisation with cell organelles, and their radiosensitising efficacy. This set was applied to the case of gadolinium-based nanoparticles (GdBN) used to amplify the radiation killing of U87 glioblastoma cells extracted from highly aggressive human tumor. For the first time, Synchrotron Radiation Deep UV (SR-DUV) microscopy is proposed as a new tool to track label-free GdBN. It confirmed the localisation of the NPs in the cytoplasm of U87 cells and the absence of NPs in the nucleus. In a second step, Transmission Electron Microscopy (TEM) demonstrated that GdBN penetrate cells by endocytosis. Third, using confocal microscopy it was found that GdBN co-localise with lysosomes but not with mitochondria. Finally, clonogenic assay measurements proved that the presence of NPs in the lysosomes induces a neat amplification of the killing of glioblastoma cells irradiated by gamma rays. The set of combined experimental protocols—TEM, SR-DUV and confocal microscopy—demonstrates a new standard method to study the localisation of label-free NPs together with their radiosensitising properties. This will further the understanding of NP-induced radiosentisation and contribute to the development of nanoagents for radiotherapy.

## Background

Glioblastoma multiforme (GBM) is a highly aggressive tumor with median survival time of patients of 12 months [1]. Hence, the treatment of this type of cancer remains a challenge. Recently, high-Z atoms containing nanoparticles (NPs) have been proposed as potential nanodrugs to improve the effects of radiation-based therapies [2–5]. Among metal-based NPs, gold NPs were widely used for diagnostic as contrast agents and in therapy [6–10]. Gold NPs were found to enhance the effects of mid- and high-energy X-rays [2,4–6,11]. In parallel, it was shown that NPs composed of other metals such as platinum are able to enhance lethal damage to biomolecules when gamma rays or fast medical ions (He^2+^ and C^6+^) are used as ionising radiations [12].

Gadolinium-based nanoparticles (GdBN) act as multimodal agents; it offers a strong advantage to improve not only the therapeutic index of the treatment but also the diagnosis of tumor by MRI (theranostic) [13–16]. Importantly, *in vivo* experiments demonstrated that these NPs are rapidly eliminated by the kidneys and show no evidence of toxicity (no perturbation of the complement system, no impairment of the renal function) [17–20]. It was found that GdBN amplify significantly radiation-induced killing of U87 glioblastoma cells when combined with high-energy X-rays and gamma rays [21,22], or with fast ions [23].

The amplification effects induced by high-Z NPs are explained in terms of early stage processes that take place in the cells. Briefly, when activated by the incident radiation, NPs are responsible for the emission of electron bursts and the production of radical clusters (reactive oxygen species). Consecutively highly lethal nano-sized damages are induced in cell constituents as due to the interaction of the highly reactive clusters with biomolecules [24,25].

So far it was shown that platinum compounds (NPs or salts), gold nanoparticles and GdBN amplify cell killing even though they do not enter cell nuclei [23,26–30]. Experiments focused on the NPs localisation were conducted using Transmission Electron Microscopy (TEM) and/or confocal microscopy as standard methods. TEM allows high resolution (10 nm) observation of cell organelles such as liposomes and mitochondria. The limitation of this technique, however, stems from the difficult sample preparation, which may change the morphology of the cells [31]. In confocal microscopy, the experiments are performed with living cells, thus measurements of the uptake dynamic and co-localisation with cell organelles (lysosomes, mitochondria) can be performed. The limitation of confocal microscopy stems from the necessity to tag the NPs with fluorescent dyes such as rhodamine, cyanine, or BoDIPYs [32]. These markers may influence the internalisation and the localisation of the NPs in the cells. Moreover, if the dyes separate from the NPs, the fluorescent images may lead to false interpretation [33–35].

The main goal of the present work is to determine the localisation and related radiosensitising properties of *label-free* GdBN in human glioblastoma cells (U87) in different conditions of incubation. The localisation of label-free NPs was performed with a novel microscopy tool, the Synchrotron-Radiation Deep UV (SR-DUV) microscopy. The excitation window of the synchrotron source goes down to 190 nm. The instrument is thus able to excite and detect the natural fluorescence of nanoparticles that absorb in the Deep-UV spectral range (below 350 nm). This microscope has been used to follow the intake of antibiotics in bacteria [36]. Here, we show for the first time that the technique can be applied in the observation of label-free NP uptake in cells. As a complementary tool, TEM was used to characterise the uptake mechanism of GdBN in U87 cells. Additional measurements using confocal microscopy were used to follow the dynamics of NPs in *living* cells and also to co-localise the GdBN with lysosomes and mitochondria, two important organelles in cell metabolism. The effect of GdBN on the radiation induced cell killing of U87 glioblastoma cells irradiated by gamma rays 1.25 MeV was evaluated using clonogenic assay [22].

## Methods

*Gadolinium-based nanoparticles (GdBN)* were synthesised by the group of O. Tillement (LPCML, Lyon, France) [13]. Briefly, the GdBN consist of a polysiloxane core surrounded by gadolinium chelates covalently grafted on the inorganic matrix. The synthesis procedure and the characteristics of these nanoparticles are detailed elsewhere [22,37]. Their size is 3 ± 1 nm in diameter and their mass is about 8.5 ± 1.0 kDa [22,37]. After freeze-drying, the GdBN can be stored at 4°C for months. For SR-DUV microscopy and TEM measurements, label-free GdBN were used. For the experiments of confocal microscopy, the organic fluorophore cyanine 5.5 was covalently grafted onto GdBN [15,22,38]. The concentration of GdBN in the medium is expressed in the concentration of Gd, *i. e.*, 1 mM of Gd is approximately equal to 0.1 mM of nanoparticles. In this study we used Gd concentrations ranging from 0.5 to 2 mM, which are not toxic for U87 cells [30].

### Cell culture

Human glioblastoma U87 cells were cultivated in Dulbecco’s Modified Eagle Medium (DMEM) (Life Technologies) supplemented with 10% heat-inactivated foetal bovine serum (PAA), 100 U/ml penicillin (PAA), 100 μg/ml streptomycin (PAA) and 1% Non Essential Amino Acids (Life Technologies).

### SR-DUV fluorescence microscopy

Cells were plated on quartz slides (ESCO OPTICS Inc) and maintained in 5% CO_2_ incubator at 37°C. Medium containing GdBN at 0.5 mM or 2 mM concentration was added to the cells during 5 min or 1 hour. After the incubation, cells were rinsed twice with PBS 1X (5 min at room temperature), fixed with 4% paraformaldehyde in PBS 1X (20 min at room temperature), rinsed with distilled water, dried and stored at 4°C. The SR-DUV experiments were performed at the DISCO beamline of Synchrotron SOLEIL (Saint-Aubin, France).

The fluorescence images were recorded at λ_exc_ = 340 nm. The acquisition time of one image was 30 s. Images of minimum three cells were recorded for each condition including the control. Sixty images with a vertical z-stack of 0.25 μm were acquired to obtain a 3D record of the GdBN localisation in the cells. The Tikhonov-Miller deconvolution was applied to correct the distortion of the images. The images were finally analysed with ImageJ software (Rasband, W.S., ImageJ, U. S. National Institutes of Health, Bethesda, Maryland, USA, http://imagej.nih.gov/ij/, 1997–2011). The total fluorescence intensity of each cell was determined using ImageJ (integrated density, ID). The background was obtained by measuring the fluorescence intensity of regions out of the cells. The corrected total cell fluorescence (CTCF) was then determined by subtracting the background from the integrated density (CTCF = ID – background).

### Transmission electron microscopy (TEM)

It was performed on the microscopy platform IBiSA at the Institut Curie, Orsay, France. U87 cells were plated on microscopic glass slides. The samples were incubated with 1 mM GdBN during 1 hour. The slides were then rinsed with PBS 1X and fixed with a mixture of 2.5% glutaraldehyde and 4% paraformaldehyde diluted in PBS 1X. After rinsing with PBS 1X, the cells were dehydrated using ethanol in gradient concentrations and embedded step by step in Epon resin. After resin polymerisation, the samples were cut using an ultramicrotome in 100 nm-thick slices. The ultrathin sections were deposited on carbon-formvar copper grids (Agar scientific) and observed under Z-loos mode (10 eV window) in a JEOL 2200FS electron microscope operated at 200 kV. Measurements were performed for close to 20 U87 cells of 4 different slices. Additional Electron Energy Loss Spectroscopy (EELS) measurements were performed using an omega-filter.

### Confocal microscopy studies

The experiments were performed with a LEICA SP5 confocal system located at the Centre de Photonique Bio-Medical (CPBM), University Paris Sud, Orsay, France. The samples were thermostatically controlled and regulated in CO_2_. U87 cells were grown in 8-well LabTek chambers (Nalge Nunc International). For simple localisation studies, the cells were incubated with 0.6 or 1 mM of GdBN functionalised with cyanine 5.5 used as a fluorescent marker (GdBN-Cy5.5) during different incubation times (1 hour, 6 hours and 12 hours). After incubation the cells were rinsed three times with PBS 1X and Hank’s Balanced Salt Solution (HBSS) was added. Cyanine 5.5 was excited at 633 nm and the fluorescence emission was detected in the 650-750 nm range. The localisation was studied in more than 100 cells.

For co-localisation studies, U87 cells were incubated with Lysotracker-green (Invitrogen) (75 nM) or Mitotracker-green (Invitrogen) (200 nM) dissolved in HBSS and mixed with DMEM for 45 minutes. The trackers were washed out with PBS 1X before incubation during 1 hour with 1 mM of GdBN-Cy5.5. After incubation the cells were rinsed three times with PBS 1X and Hank’s Balanced Salt Solution (HBSS) was added. Cyanine 5.5 was excited at 633 nm and the fluorescence emission was detected in the 650-750 nm range. The lysotracker and mitotracker were excited at 488 nm and the fluorescence emission was detected in the 505-600 nm range. Images were recorded at three different depths (z axis-positions). The co-localisation of GdBN with lysosomes and mitochondria was evaluated using the ImageJ software and the JACoP statistical plug-in (Just Another Co-localisation Plugin) (http://rsb.info.nih.gov/ij/plugins/track/jacop.html). The co-localisation coefficients were calculated for more than 30 cells.

### Gamma radiation experiments

1.5 ×10^5^ exponentially growing U87 cells were plated in Petri dishes (Falcon 3002) 12 hours before irradiation. Cells were maintained in a 5% CO_2_ incubator at 37°C. GdBN were added to the cell medium 6 hours before irradiation at a gadolinium concentration of 0.5 mM. At this concentration, the nanoparticles are not toxic [19,20]. The U87 cells were irradiated at room temperature under atmospheric conditions. The irradiations were performed with a cobalt source (^60^Co) at CEA (Fontenay aux Roses, France) at a mean energy of 1.25 MeV, a linear energy transfer (LET) of 0.2 keV/μm and a dose rate of 1 Gy/min. The combined effect of radiation and nanoparticles on cells was quantified by clonogenic assay. After irradiation, the cells were fixed by trypsin and plated into 100 mm Petri dishes (Falcon 3002) at a density of 100 surviving cells per dish. The plating efficiency was found close to 20%. After 14 days, the colonies were treated with 50% methanol and stained by 0.5% methylene blue. The colonies were counted to determine the survival fractions.

## Results and discussion

### Localisation of *label-free* GdBN in U87 cells with SR-DUV microscopy

**Figure 1:**
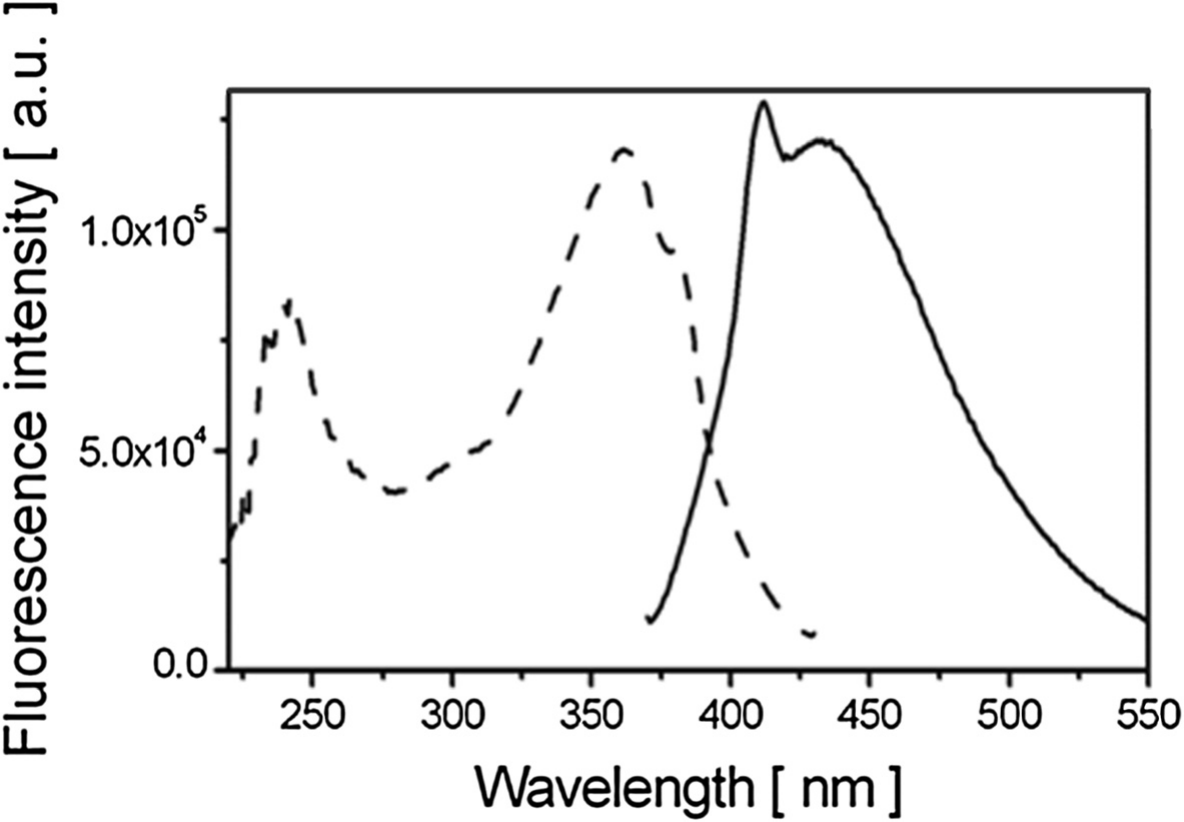
**Fluorescence spectra of label free GdBN.** Fluorescence excitation spectrum (**λ**_em_ = 440 nm) (- -) and fluorescence emission spectrum (**λ**_exc_ = 360 nm) (**-**) of 1 mM GdBN.

In a first step, the spectroscopic properties of GdBN (1 mM) were determined by performing the fluorescence excitation spectroscopy and the fluorescence emission spectroscopy of NPs diluted in ultra-pure water (Figure 1). GdBN exhibited the maximum fluorescence emission at **λ**_em_ = 440 nm and the maximum excitation at **λ**_exc_ = 360 nm.

In cells, an autofluorescence is generated by natural fluorophores, mainly NADH, tyrosine, and tryptophan [39]. To obtain the best signal of the NPs over the autofluorescence, the excitation wavelength **λ**_exc_ = 340 nm was chosen for all the microscopy experiments.

**Figure 2:**
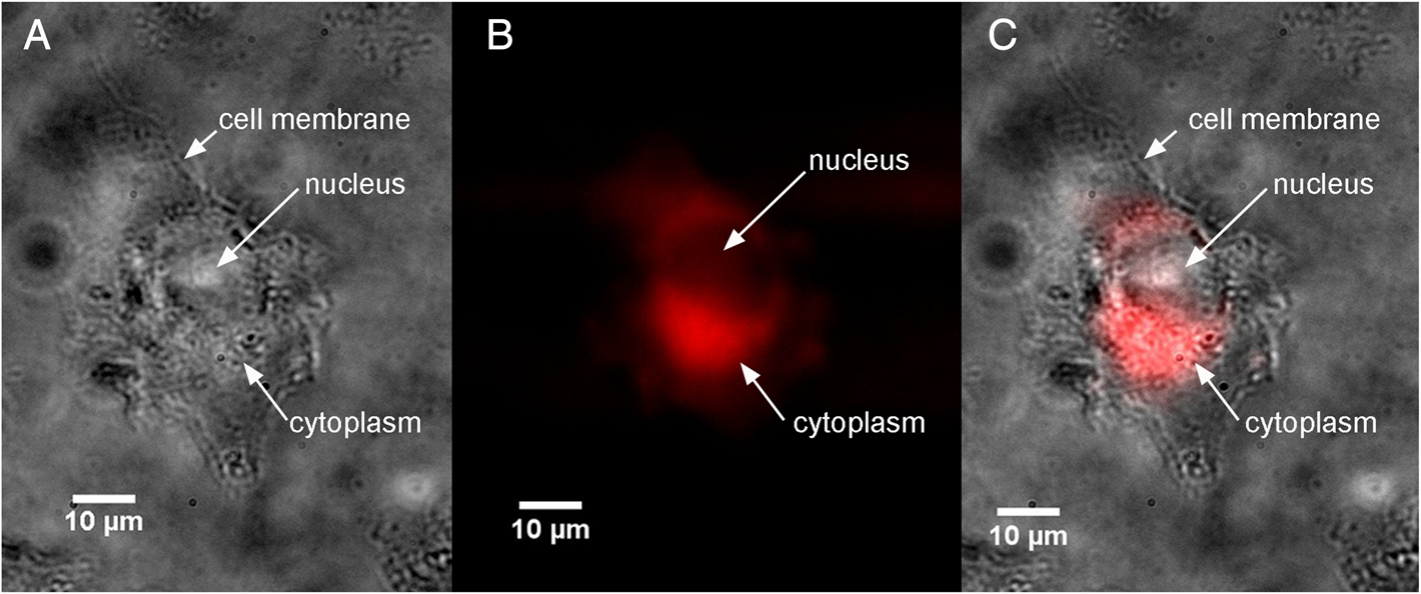
**Localisation of GdBNs in U87 cells visualised by SR-DUV microscopy. (A)** Light transmission image of U87 cell, **(B)** fluorescence image of label free GdBN (red), **(C)** merge of transmission and fluorescence images (GdBN in red).

In a second step, the fluorescence microscopy of GdBN in U87 cells was recorded. The experiments were performed with cells incubated with GdBN at two concentrations (0.5 and 2 mM) during 5 minutes or 1 hour. The result obtained for the GdBN concentration of 2 mM and the incubation time of 5 min is presented in Figure 2. Light transmission micrograph was used to display the shape of the cells (Figure 2A). The nucleus of the cell was clearly distinguished as pointed in the figure. This image shows that the cell did not suffer from the sample preparation. Figure 2B corresponds to the SR-DUV fluorescence image. It shows that GdBN were present. The merge of Figures 2A and B (Figure 2C) is used to show the localisation of GdBN in cells. We clearly observe that GdBN *free of fluorescent dye* enter the cells and remain located in the cytoplasm exclusively.

In order to investigate the influence of the GdBN concentration and incubation time on NPs uptake, we calculated the corrected total cell fluorescence (CTCF) values for cells free of GdBN (controls) and cells loaded with two concentrations (0.5 and 2 mM) for the two incubation times (5 min and 1 hour). The CTCF values are summarised in Table 1.

**Table 1:** **CTCF values measured in U87 cells with and without NPs**

**GdBN concentration [mM]**	**GdBN incubation time**	**Mean CTCF**	**CTCF cell 1**	**CTCF cell 2**	**CTCF cell 3**	**CTCF cell 4**
2	5 minutes	**3.88 10** ^**7**^	7.58 10^7^	1.65 10^7^	4.15 10^7^	2.12 10^7^
2	1 hour	**2.84 10** ^**7**^	2.68 10^7^	1.33 10^7^	2.19 10^7^	5.16 10^7^
0.5	1 hour	**2.86 10** ^**7**^	4.77 10^7^	1.13 10^7^	2.68 10^7^	
0 (control)		**1.70 10** ^**7**^	1.36 10^7^	2.14 10^7^	1.60 10^7^	

The present analysis shows that there was a high variability of fluorescence intensity between the different cells. This indicates that the uptake was not homogeneous in every cell. Interestingly, GdBN are already efficiently internalised in the cells after 5 minutes. However the concentration of GdBN in the medium (0.5 mM and 2 mM) did not impact the quantity of GdBN in the cells. This last result seems different from the measurements of Rima and coworkers who observed a linear increase of the Gd concentration in U87 and SQ20B cells whilst increasing the GdBN concentration in the cultivation medium [30]. However their study was performed using the Inductively Coupled Plasma (ICP) technique, which consists of the quantification of internalised Gd averaged over millions of cells. Contrary to this macroscopic technique, SR-DUV microscopy gives rise to the internalisation of GdBN in each cell. This allows the observation of differences between cells as observed in the present study. This type of cell-to-cell heterogeneity was recently observed in the case of magnetic NPs [40]. The heterogeneity in the capacity of cells to internalise NPs could influence their efficacy of amplifying radiation effects. It is thus a great challenge to study and quantify the heterogeneity in the uptake of label free-NPs which has never been done so far. This possibility offered by SR-DUV microscopy is still in the process of development.

### Uptake mechanism of *label-free* GdBN in U87 cells investigated by TEM

TEM measurements have been performed to observe the internalisation of NPs with a better resolution (10 nm) and determine the mechanism(s) of NPs uptake.

**Figure 3:**
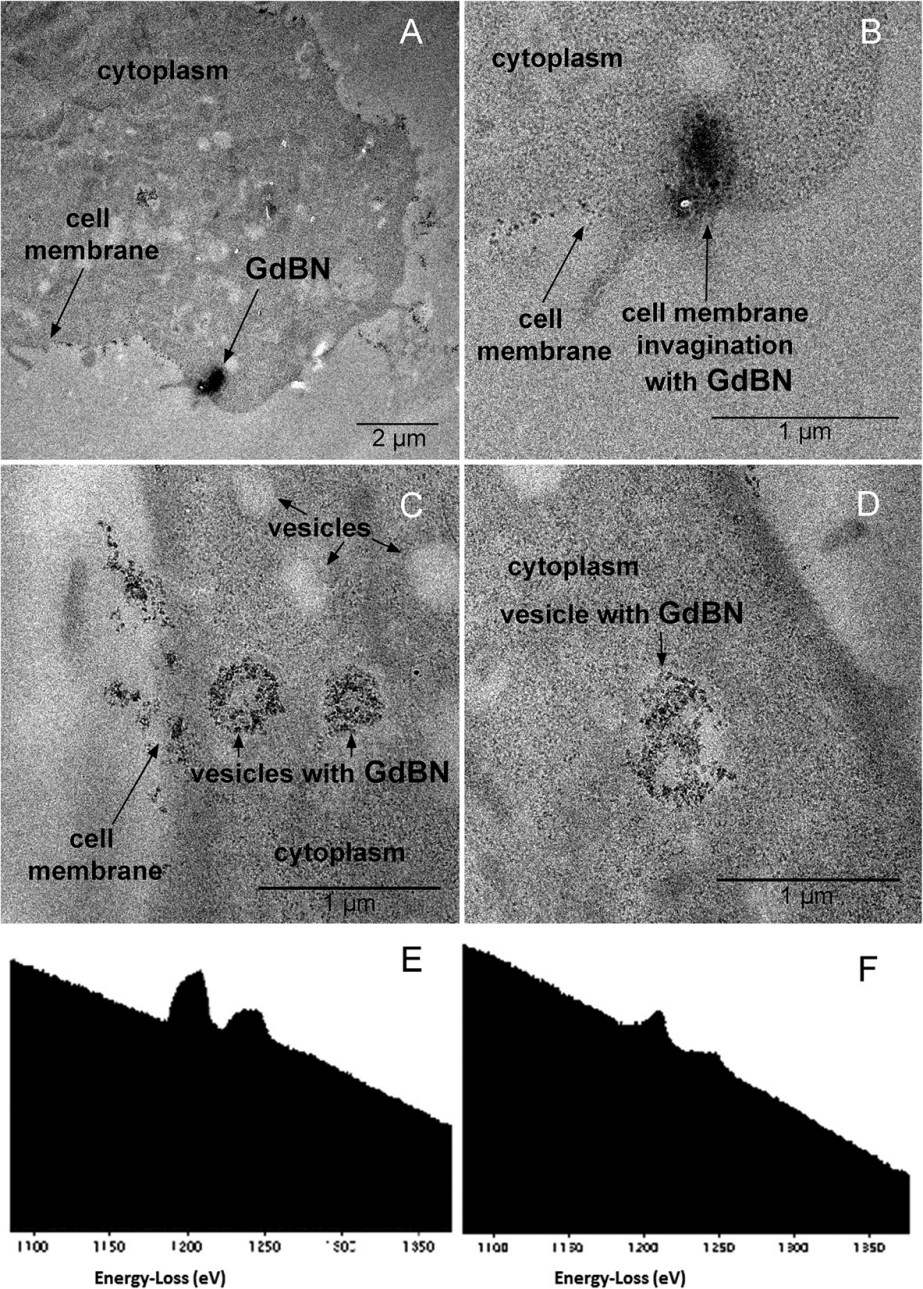
**TEM images of U87 cells after incubation with 1 mM GdBN for 1 hour. (A)** Image of a cell with electron dense regions located close to the membrane. **(B)** Zoom of the electron dense region shown in A. **(C and D)** Images of cells with electron dense regions located in the cytoplasm. **(E)** EELS spectrum of a electron dense region evidenced in (A). **(F)** EELS spectrum of a electron dense region evidenced in **(C)**.

Images have been recorded for more than 20 samples (see Experimental section). An example of TEM image is presented in Figure 3. For all the samples we observe electron dense regions close to the cell membrane (Figure 3A and 3B) and in the cytoplasm (Figure 3C and 3D). The magnification image (Figure 3B) shows that these regions are composed of small electron dense objects. These are attributed to nanoparticles clusters. No electron dense regions were found in the cell nucleus (image not shown here).

To confirm the composition of these dense granules, EELS spectra of these regions have been performed close to the membrane and in the cytoplasm (Figure 3E and 3F). The peaks M4 and M5 are characteristic of gadolinium. Finally, these TEM and EELS measurements confirm that GdBN entered U87 cells and located in the cytoplasm but do not penetrate the cell nuclei.

Interestingly NPs clusters appear in low electron density regions. These regions are assigned to vesicles. The average diameter of these vesicles is 400 – 600 nm, which corresponds to endosomes and lysosomes [41]. In the images 3A and 3B we also observe the presence of membrane invaginations. These observations strongly suggest that GdBN were internalised by endocytosis. Indeed, this uptake mechanism is characterised by the induction of membrane invagination followed by trafficking from early endosomes to late endosomes and lysosomes as observed in the TEM images.

It is noteworthy to mention that GdBN have a size (3 nm) close to macromolecules such as proteins. These entities are generally transported to cells by pathways such as phagocytosis, macropinocytosis, clathrin-dependent endocytosis, caveolin-dependent endocytosis and clathrin/caveolinin dependent endocytosis [42,43]. These mechanisms differ by the size of the vesicles, the nature of the transported species and the need of specific receptors [44]. Rima and co-workers [30] observed that, in the case of SQ20B cell line, GdBN are internalised *via* macropinocytosis. This process is characterised by the formation of membrane lamellipodia (“arms”) which pick up particle aggregates [30]. In the present study, the presence of membrane invaginations is more indicative of a pathway such as clathrin-mediated endocytosis. The uptake of GdBN by endocytosis has been proposed by other groups [45,46]. In the case of nanodiamonds internalised in A549 lung cancer cells, it was shown that different pathways such as macropinocytosis and clathrin-mediated endocytosis may participate in the NPs uptake [47]. Similarly, various endocytosis mechanisms were used to describe the uptake of sub-20 nm titanium dioxide nanoparticles in prostate PC-3 M cancer cells [48].

Finally, the present study shows that in the case of U87 glioblastoma cells, GdBN were internalised by endocytosis. It is not clear yet what are the parameters that give advantage to one or more uptake pathways and how important this step is in the impact of NPs on the radiation effects. Additional studies are needed to answer these questions.

### Influence of fluorescent labelling on NPs uptake and co-localisation with cell organelles performed by confocal microscopy

The other objective of this work was to co-localise GdBN with lysosomes and mitochondria of U87 cells. For this purpose, fluorescent labelling of GdBN was needed because confocal microscopy is the standard method used to image cell organelles. We first investigated the influence of cyanine 5.5 on the NPs localisation to prevent artefacts due to the presence of fluorescent dyes in the co-localisation measurements.

#### Influence of cyanine 5.5 on the GdBN localisation in U87 cells.

**Figure 4:**
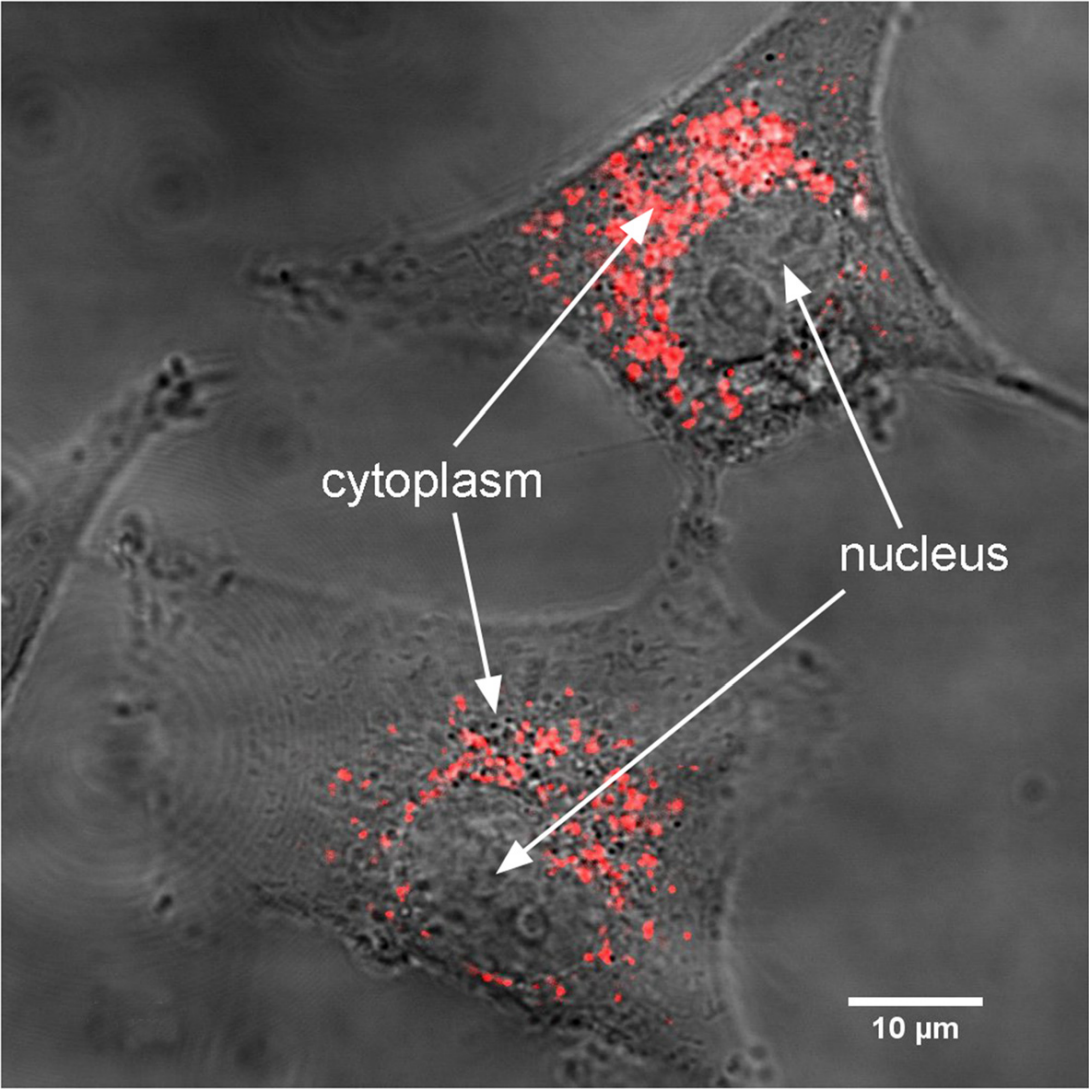
**Merge image of the transmission and fluorescence images obtained by confocal microscopy of U87 cell loaded with GdBN-Cy5.5 (red) at a concentration of 0.6 mM incubated for 12 hours.**

A representative fluorescent image of U87 cells loaded with GdBN tagged with cyanine 5.5 (GdBN-Cy5.5) is presented in Figure 4. This image confirmed that the NPs sit exclusively in the cytoplasm even in the presence of cyanine at the surface. Similar results were obtained for different conditions of incubation (GdBN concentrations and incubation times – see section Methods). Interestingly, NPs did not enter the nucleus but were located around the nuclei. This is in agreement with results obtained with GdBN localised in Chinese Hamster Ovary (CHO) cells [23] and U87 cells [22].

The GdBN clusters have a size distribution in the cell cytoplasm ranging from 400 to 900 nm. This distribution was stable regardless of the NPs concentration (0.6 or 1 mM) and the incubation time (1 hour, 6 hours and 12 hours). When GdBN are conjugated with fluorescein- isothiocyanate (FITC) the clusters are larger and more irregular [22].

It is worth mentioning that GdBN remained localised in the cells for up to 37 hours (end of our observation), which indicates that the NPs had a long time of residence in U87 cells.

More importantly the localisation of GdBN tagged with cyanine 5.5 was similar to the localisation of label-free NPs as observed in TEM and SR-DUV microscopy. Thus, we have demonstrated without ambiguity that the addition of cyanine 5.5 did not influence the localisation of GdBN in U87 cells. In conclusion, confocal microscopy together with NPs labelling with cyanine can be used to perform additional measurements of co-localisation with cell organelles.

The novel methodological approach used here—combining TEM, SR-DUV and confocal microscopy—is proposed for other studies focused on the NPs localisation in cells.

#### Co-localisation of GdBN with cell organelles

In a second step, we investigated the co-localisation of GdBN with lysosomes and mitochondria with confocal microscopy using cyanine as the fluorescent dye of the NPs.

**Figure 5:**
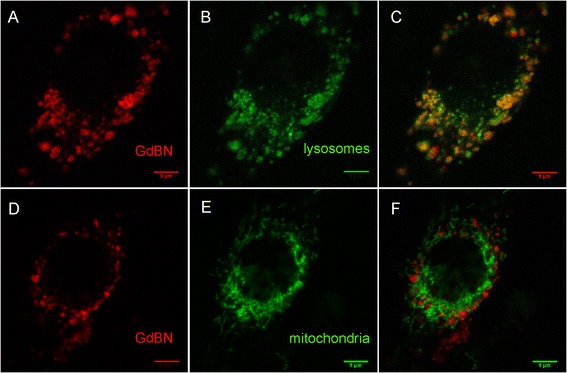
**Fluorescence images obtained by confocal microscopy of U87 loaded with GdBN-Cy5.5 1 mM (red) (A, D, C and F) in the presence of Lysotracker-green (green) (B and C) or Mitotracker-green (green) (E and F).** **(C)** Merged image of **(A)** and **(B)**. **(F)** Merged image of **(D)** and **(E)**.

Confocal microscopy images are presented in Figure 5. Figure 5A and 5D correspond to the fluorescence images of U87 cells loaded with GdBN-Cy5.5. Figure 5B and 5E correspond to the fluorescence images of U87 cells incubated with Lysotracker-green or Mitotracker-green respectively. The merged images of GdBN-Cy5.5 with Lysotracker-green (Figure 5C) and Mitotracker-green (Figure 5F) clearly demonstrate the co-localisation of GdBN-Cy5.5 with lysosomes but not with mitochondria.

A statistical analysis of the images was performed using ImageJ statistical plugin JACoP, a tool commonly used for co-localisation analysis. Briefly, this plugin gives access to the most important correlation coefficient-based tools (Pearson’s coefficient, Manders’ coefficient) and allows comparing various methods (Costes’ approach, Van Steensel’s approach, Li’s approach) to evaluate the co-localisation [49]. The Pearson’s correlation coefficient was used to quantify the correlation between the GdBN-Cy5.5 fluorescence and the lysosomes or mitochondria fluorescence. This correlation coefficient estimates the degree of overlap of the red and green spots of each dual-channel image [50]. This analysis was performed with the images recorded 20 hours after the incubation with GdBN-Cy5.5. The average Pearson’s correlation coefficients obtained were 0.63 (SD 0.078) for the GdBN co-localisation with the lysosomes and 0.23 (SD 0.091) for the GdBN co-localisation with the mitochondria. Values in the 0.5 - 1 range indicate a co-localisation [51]. This analysis demonstrates that GdBN were co-localised with lysosomes. This was observed for 5 hours up to 37 hours after incubation. No co-localisation of GdBN with mitochondria was observed even after 37 hours.

Finally, the present experiment displayed lysosomes as being the preferential sites of GdBN in U87 cells.

It should be noted that lysosomes are very acidic cellular vesicles that play a role in the transport and the degradation of intracellular and extracellular cargo. A perturbation of these entities (by radiation for instance) can induce lysosomal pathologies such as phospholipidosis, lysosomal overload, which result in the autophagy of the cells [52]. The presence of NPs of different sizes and composition in endosomes and lysosomes was shown by other groups [53–56].

### Influence of GdBN on gamma radiation effects in U87 cells

**Figure 6:**
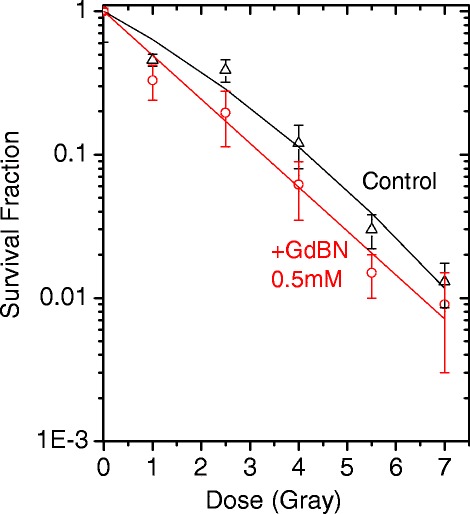
**Surviving fraction as function of radiation dose of U87 cells free of GdBN (black) and in the presence of GdBN (red) irradiated by gamma rays (** ^**60**^ **Co).**

The effect of GdBN on cells irradiated by gamma rays (1.25 MeV) was investigated using the clonogenic assay as the analysis method of the radiation-induced cell killing (see Methods section). The survival curves of U87 cells free of nanoparticles (controls) and U87 cells loaded with GdBN (0.5 mM) irradiated by gamma rays are presented in Figure 6.

The cell survival fractions decreased while the radiation dose increased. This decrease was clearly amplified in the presence of GdBN.

To characterise the effects of the nanoparticles, the cell surviving fraction (SF) curves were simulated with a linear quadratic law [57]:

1 $$ SF(D)={e}^{-\left( aD+\beta {D}^2\right)} $$

where D is the irradiation dose. The coefficient α corresponds to the contribution of lesions, which are directly lethal for the cell and β is attributed to the contribution of additive sub-lethal lesions. The values of α and β determined by a fitting procedure are reported in Table 2.

**Table 2:** **Coefficients α and β calculated for U87 cells irradiated by gamma rays**

	**α (Gy** ^**−1**^ **)**	**β (Gy** ^**−2**^ **)**
Control	0.4 ± 0.1	0.03 ± 0.02
GdBN	0.71 ± 0.03	0

**Table 3:** Surviving fraction (SF) and enhancing factor (EF) calculated for U87 cells irradiated by gamma rays

	**SF** ^**2 Gy**^	**EF**
Control	0.31	
GdBN	0.24	22.6%

This analysis shows that the presence of GdBN induces an increase of directly lethal lesions (α) and a decrease of sub-lethal lesions (β). The enhancement of directly lethal lesions is attributed to the amplification of complex molecular damage as shown elsewhere [23].

The efficiency of GdBN to amplify radiation-induced cell death was characterised by calculating the enhancing factor (EF) at 2 Gy:

2 $$ EF=\frac{\left(S{F}_{control}^{2 Gy}-S{F}_{GdBN}^{2 Gy}\right)}{S{F}_{control}^{2 Gy}} $$

The survival fractions at 2 Gy of U87 cells free of NPs (*SF*_*control*_^2*Gy*^) and loaded with GdBN (*SF*_*GdBn*_^2*Gy*^) are 0.31 and 0.24, respectively. The enhancing factor is close to 23%, which characterises the GdBN efficiency when 1.25 MeV gamma rays are used as ionising radiation (see Table 3). These results are complementary to the other studies performed with GdBN and summarised in reference [58].

## Conclusions

The main objective of this work was to probe the efficacy of NPs to amplify radiation effects as a function of their localisation in the cells. We found that GdBN efficiently amplify gamma rays-induced cell killing of U87 cells (by 23%) even though NPs are located and activated in lysosomes but not in mitochondria. It is the first evidence that the radiosensitisation by NPs may be due to strong perturbations in lysosomes.

This work required the optimisation of an experimental protocol based on the combination of three techniques with the aim to determine unambiguously the localisation of label-free NPs. The synchrotron radiation deep-UV (SR-DUV) microscopy was proposed as a new tool to observe the uptake of *label-free* NPs that do not absorb in the visible energy range. This elegant technique offers new perspectives in cell microscopy. Conventional TEM was used to determine the uptake mechanism of GdBN. Finally, confocal microscopy was used to investigate the co-localisation of NPs with cell organelles with no artefact of the Cyanine dye in this case. With this set of methods, we demonstrated that GdBN are taken up by U87 cells via endocytosis and start to populate lysosomes 5 hours after incubation but never reach the mitochondria. Thus we demonstrated that the amplification of radiation effects by GdBN in U87 cells is related to perturbations induced in the cell lysosomes but not in the respiratory chain system (in the mitochondria). This study is a first step in the understanding of the biological action of GdBN in U87 cells; additional experiments are needed to identify the metabolic functions that are affected by the presence and the activation of NPs in lysosomes.

Finally, the study demonstrates that a combination of experimental protocols—TEM, SR-DUV and confocal microscopy—may be used as a standard method to characterise the action of NPs in different cell lines.

## Abbreviations

CEA: Atomic energy center
CHO: Chinese hamster ovary
CPBM: Centre de photonique bio-medical
CTCF: Corrected total cell fluorescence
DMEM: Dulbecco’s modified eagle medium
GBM: Glioblastoma multiforme
GdBN: Gadolinium-based nanoparticles
GdBN-Cy5.5: GdBN labelled with cyanine 5.5
HBSS: Hank’s balanced salt solution
EELS: Electron energy loss spectroscopy
EF: Enhancing factor
FITC: Fluorescein IsoThioCyanate
ICP: Inductively coupled plasma
LET: Linear energy transfer
NPs: Nanoparticles
SD: Standard deviation
SF: Surviving fraction
SR-DUV: Synchrotron-radiation deep UV
SQ20B: Human head and neck squamous cells carcinoma cell line
TEM: Transmission electron microscopy
U87: Human glioblastoma cell line
